# Adult *Drosophila melanogaster *evolved for antibacterial defense invest in infection-induced expression of both humoral and cellular immunity genes

**DOI:** 10.1186/1756-0500-4-305

**Published:** 2011-08-23

**Authors:** Yixin H Ye, Elizabeth A McGraw

**Affiliations:** 1 School of Biological Sciences, Monash University, Melbourne, Vic 3800, Australia

## Abstract

**Background:**

While the transcription of innate immunity genes in response to bacterial infection has been well-characterised in the Drosophila model, we recently demonstrated the capacity for such transcription to evolve in flies selected for improved antibacterial defense. Here we use this experimental system to examine how insects invest in constitutive versus infection-induced transcription of immunity genes. These two strategies carry with them different consequences with respect to energetic and pleiotropic costs and may be more or less effective in improving defense depending on whether the genes contribute to humoral or cellular aspects of immunity.

**Findings:**

Contrary to expectation we show that selection preferentially increased the infection-induced expression of both cellular and humoral immunity genes. Given their functional roles, infection induced increases in expression were expected for the humoral genes, while increases in constitutive expression were expected for the cellular genes. We also report a restricted ability to improve transcription of immunity genes that is on the order of 2-3 fold regardless of total transcription level of the gene.

**Conclusions:**

The evolved increases in infection-induced expression of the cellular genes may result from specific cross talk with humoral pathways or from generalised strategies for enhancing immunity gene transcription. A failure to see improvements in constitutive expression of the cellular genes suggests either that increases might come at too great a cost or that patterns of expression in adults are decoupled from the larval phase where increases would be most effective. The similarity in fold change increase across all immunity genes may suggest a shared mechanism for the evolution of increased transcription in small, discrete units such as duplication of *cis*-regulatory elements.

## Introduction

Recently, using selection experiments, we examined the ability of *Drosophila melanogaster *to evolve in response to systemic infection by the opportunistic bacterial pathogen, *Pseudomonas aeruginosa *[1]. The evolved flies demonstrated an improved defense as measured by an increase in fly survival in response to infection from 15 to 70%. The genetic basis of the evolved response was examined using microarrays and was characterised by increased expression for many immunity related genes. The evolved defense was costly, as evidenced by decreases in egg viability and longevity and the rapid loss of the increased survival when selection was removed. While the original study demonstrated the involvement of both cellular and humoral aspects of insect immunity in the evolved defense, the design did not allow for the partitioning of the total transcriptional change into its constitutive versus infection-induced components. These two avenues for transcriptional change should have different energetic or pleiotropic costs and [2] may be more or less effective in improving immunity depending on the functional role of the gene. The original study also only focused on genetic changes in males and so did not determine if females were arriving at their improved defense by the same genetic mechanism.

The humoral immune response in insects is characterized by cascades of genes whose expression is controlled by a range of recognition proteins that bind pathogen cell wall components and end with the production of a range of secreted host proteins that target bacteria for destruction [3]. Exposure to infection can increase the transcription of these genes substantially, ie from 10-100 fold for some of the genes encoding antimicrobial peptides [4]. We hypothesized that selection for improved defense would lead to further increases in the transcription of these genes upon exposure to infection. This would represent a targeted strategy, producing these molecules only when needed at a point in the infection process where they normally act against invading bacteria. The transient and inducible nature of this aspect of immunity might suggest, however, that flies are already be operating this response at or near maximal transcriptional capacity, potentially limiting the size of gains we might see. Increasing the constitutive expression of these humoral genes, though more costly, could also be beneficial. A greater number of circulating antimicrobial peptides prior to exposure could confer an enhanced ability to contain or curtail infection and recent work has suggested that fly genotypes with greater constitutive expression of these genes are better able to control infection [5].

The genes that define the cellular response of immunity underpin processes like encapsulation, lytic ability and phagocytosis. The cellular response has been shown to be highly effective in early infection, including the recent suggestion that the inducible humoral response may exist only for "clean up" while the cellular response serves as the frontline of antibacterial defense [6]. The expression of these genes early in development can determine subsequent immunological traits in adults like the number and quality of circulating hemocytes [3,7]. Many of these genes may also have pleiotropic roles to play with respect to early development [8,9]. In our study, flies with an evolved defense also developed more rapidly, which may have resulted from such pleiotropy. Several key studies in recent years have highlighted the cost of investing specifically in cellular immunity with respect to various trade-offs in life history traits [6,10-12]. In contrast to the secreted products of the humoral response, we would expect that only increases in the constitutive expression of these genes would result in improved antibacterial defense. Any infection induced increases in expression should theoretically be "too late" or too slow convert to infection fighting ability [13]. Given the significant improvement in survival of selected flies, the cost of this improvement, the concomitant increase in developmental rate and the likely point of action of these proteins early in development [1], we expected to see evolution of increased constitutive expression for the cellular immunity genes.

Here we compare the expression patterns of immunity genes between evolved and unevolved flies in both the constitutive and infection-induced states. In so doing, we are able to partition the relative investment into constitutive versus infection-induced transcription of genes representing both humoral and cellular aspects of immunity due to selection and understand how investment in these different strategies may form the basis of fly immunity.

## Materials and Methods

Fly and bacterial culture and the selection regime are as previously reported [1] but are described briefly below. Female flies generated from the previous selection experiment are then screened here for immunity gene transcriptional profiles as an addition to the original study.

### Fly and Bacterial Culture

Brisbane (BNE) base stock was founded from 26 females *D. melanogaster *caught around the University of Queensland St Lucia campus in August 2006. The flies were initially treated with 0.5% penicillin and streptomycin in the diet for one generation [14] and then passaged without antibiotic on standard cornmeal diet supplemented with excess yeast for more than 10 generations before the start of the selection experiment. *P. aeruginosa *PA01 was cultured in LB medium supplemented with 100 mg ml^-1 ^ampicillin at 37°C on a rotory shaker [15]. For infection, the concentration of an overnight bacterial culture was adjusted to an optical density (OD) of 0.5 ± 0.05 measured spectrometrically at 600 nm. The culture was then diluted 100 fold using sterile LB. This OD was determined at the start of the selection experiments to achieve a fly population kill rate of 80-90%. To determine the number of bacterial cells used to infect each fly, individual flies were ground with a pestle in an Eppendorf tube with 200 μl of 10 mM MgSO_4 _and spread on LB plates supplemented with 100 mg ml^-1 ^ampicillin immediately after infection [16]. The average infective dose per fly was 3109 ± 635 bacterial cells.

### Selection Regime and Measurement of Defense

The base stock population of flies was split into 3 selected and 3 unselected lines. Selected lines were infected each generation with *P. aeruginosa *PA01 and the survivors allowed to populate the subsequent generation. Selection was applied for 10 generations. For each round of selection, 8 sub-replicate populations consisting of 20 flies per sex were infected with *P. aeruginosa*. Mated flies aged to 4-7 days old were anaesthetized with CO_2 _and infected as previously described by dipping a sterile needle in the bacterial culture and piercing the intrathoracic region of the fly [17]. Fly mortality was then monitored for each population over 48 hours. Survivors from each of the 8 sub-replicates were pooled into a single population to seed the subsequent generation. The unselected lines were not infected during the breeding regime, but were exposed to the same bottleneck in population size as their paired selected lines by randomly selecting a set of individuals to found the next generation. Survival in response to infection was monitored each generation in selected lines and at two time points, G_6 _and G_10 _in the unselected lines. While survival rates in the selected lines rose rapidly from 15% at G_0 _to 70% by G_10_, survival rates of unselected lines remained low. Selection was then removed at G_10 _for 5 generations and survival of the selected lines reverted to pre selection levels [1].

### Immunity Gene Expression at G_10 _after selection

There were three replicate lines in the original selection experiment (S1-S3) that all exhibited similar levels of antibacterial defense after selection. For this study we have focused on just one of the lines (S1) and its paired unselected line (C1). At G_10_, 4 to 7 day old whole female flies from these two lines were either collected directly for constitutive measures of expression or first infected with *P. aegurinosa *to measure infection-induced expression. For the infected treatment, flies were collected 8 hours post infection with *P. aeruginosa*. Five pools of 20 flies each were collected to represent each treatment for all combinations of selected/unselected × infected/uninfected. All flies were snap frozen in liquid nitrogen and extracted for Total RNA using Trizol (Invitrogen Corp., Carlsbad, CA) as per the manufacturer's instructions. Total RNA was treated with 2 μl of DNase I (Roche) for 30 minutes at 37°C in a 20 μl reaction to eliminate genomic DNA.

Real-time PCR (RT-PCR) was used to measure the expression of a set of 8 genes identified from our previous microarray study as they were shown to be involved in improved defense to *P. aeruginosa *infection in male flies [1]. Four of the genes represent the humoral (DiptB, dros4, PGRP-SB1, PGRP-SD) and 4 represent the cellular (Bc, eater, Sr-CI, TepII) immune response. Primer sequences of 6 of the genes were as reported in the initial study. In addition we designed primers for PGRP-SB1 (FBtr0075348); Forward 5'-3' TTAGCTCTATCCGCCAATGC, Reverse 5'-3' CCCTTGTGATCCGACTGAAT that generated a 191 bp product and DiptB (FBtr0086621); Forward 5'-3' CTGGCATATGCTCCCAATTT, Reverse 5'-3' ATAGGGTCCACCAAGGTGCT that generated a 198 bp product. The gene *fus*, (FBgn0023441), which is involved in an epidermal growth factor receptor signaling pathway [18], was employed a reference gene (Forward 5'-3' AAAGTGGTGGAAGCAACAGG, Reverse 5'-3' CGCACACAAACTCGAAAAGA, 158 bp product) as it is was shown not to change in response to infection or selection in the original microarray analysis [1]. There is also no evidence that female expression should differ from that of males per the meta analysis of 13 studies (Male/Female ratio = 1.07, *P *= 0.21) in the Sebida database [19].

Approximately 0.5 μg of total RNA was first reverse transcribed using random primers and SuperScript III reverse transcriptase (Invitrogen) according to manufacturer's protocols. Then RT-PCR was performed on Rotor-gene 6000 (Corbett Life Science, Sydney, NSW) using Platinum^®^SYBR^®^Green (Invitrogen Inc, Carlsbad, CA) according to manufacturer's instructions. For each sample, a mastermix of 2 μl RNase-free water, 5 μl of SYBR Supermix and 0.5 μl of each primer (10 μM) was added to 2 μl of cDNA. Three technical replicates were run for each biological replicate. The cycling protocol was as follows; 1 cycle UDG incubation at 50°C for 2 minutes, 1 cycle *Taq *activation at 95°C for 2 minutes, 40 cycles of denaturation at 95°C for 5 s, annealing at 60°C for 5 s, extension at 72°C for 15 s, fluorescence acquisition at 78°C, and 1 cycle of melt curve analysis from 68-95°C in 1°C steps. Mean Cycle Threshold (CT) and mean amplification efficiency (E) per biological replicate was calculated across technical replicates using Rotor-Gene 6000 Series Software ver.1.7.75 (Info-ZIP Pty Ltd.) for analysis.

**Figure 1:**
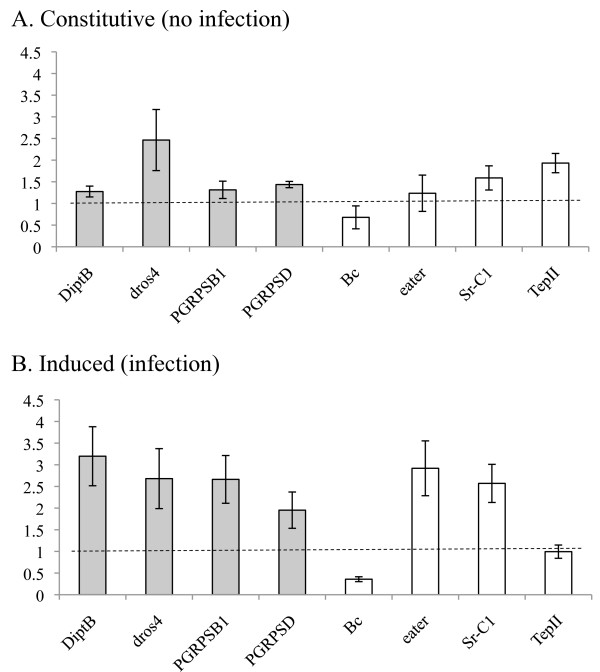
**Fold change in expression in response to selection (as opposed to total transcriptional output) in the constitutive (A) and infection induced (B) states**. Mean ratios of selected/unselected flies ± sem for random pairings of biological replicates.

Expression ratios were calculated by the Q-gene application [20]. The software transforms the logarithmic scaled raw data unit into the linear unit of normalized expression (NE) and expresses it as a ratio to the reference gene. The data were then analysed using general linear models per gene with the factors, infection status (infected vs uninfected) and treatment (selected vs unselected) using Statistica 8.0 (StatSoft, Inc.). To control the occurrence of false positive for independent test statistics, test *P*-values were corrected using the Benjamini and Hochberg method [21]. Ratios were then created of selected/unselected for display only by random pairing of replicates (Figure 1).

## Results

### Response to Selection

The transcription of all genes responded to infection and all but TepII and PGRP-SD exhibited altered transcription in response to selection (Tables 1 and 2). By comparing directly the fold change in expression due to selection in the constitutive state (Figure 1A: selected/unselected, no infection) with that in the infection-induced state (Figure 1B: selected/unselected, infection), we can see the relative investment in these two different aspects of the immune response. By examining the pattern of significance both in the main treatment effect of selection and in its interaction with infection (Tables 1 and 2), we see that most of the transcriptional change in response to selection occurs only in the presence of infection (Figure 1). Only dros4 exhibited similar levels of fold increase after selection both in the constitutive and the infection-induced states (Table 1, Figure 1). DiptB, PGRP-SB, Bc, eater and Sr-CI demonstrate significant interactions, Selection*Infection, with transcriptional changes that are only present after infection. PGRP-SD though, not statistically significant (P = 0.068), exhibits a similar trend. The changes in these genes were all in the direction of increased transcription, save Bc, that showed a greater decrease upon exposure to infection. The mean increase in infection induced transcription due to selection was modest, typically 2-3 fold (Figure 1). The impact of a 2-fold increase, however, can be quite substantial with respect to total transcription levels for very highly expressed genes such as PGRP-SB1, PGRP-SD1 and DiptB [4].

**Table 1:** Gene specific general linear models for the effects of selection and infection on expression of humoral genes

	df	DiptB		dros4		PGRP-SB1		PGRP-SD	
		**F**	**P**	**F**	**P**	**F**	**P**	**F**	**P**

Intercept	1	51.97	0.00	64.73	0.00	58.89	0.00	51.28	0.00

Selection	1	11.91	**0.0032**	8.60	**0.0097**	9.34	**0.0075**	3.80	0.068

Infection	1	50.98	**< 0.0001**	19.32	**< 0.001**	51.17	**< 0.0001**	38.58	**< 0.0001**

Selection*Infection	1	11.85	**0.0033**	2.68	0.12	8.93	**0.0086**	3.19	0.093

Error	16								

Degrees of freedom (df), F-statistics (F) and the p-value (P), bold denotes significance at P < 0.05 after Benjamini-Hochberg [21] multiple test correction.

**Table 2:** Gene specific general linear models for the effects of selection and infection on expression of cellular genes

	df	Bc		eater		Sr-CI		TepII	
		**F**	**P**	**F**	**P**	**F**	**P**	**F**	**P**

Intercept	1	54.75	0.00	109.61	0.00	210.63	0.00	73.96	0.00

Selection	1	10.14	**0.0057**	7.67	**0.013**	24.02	**0.00016**	0.11	0.73

Infection	1	15.36	**0.0012**	11.23	**0.0040**	58.26	**< 0.0001**	37.24	**< 0.0001**

Selection*Infection	1	6.37	**0.022**	7.16	**0.016**	13.99	**0.0017**	0.17	0.68

Error	16								

Degrees of freedom (df), F-statistics (F) and the p-value (P), bold denotes significance at P < 0.05 after Benjamini-Hochberg [21] multiple test correction.

### Comparison to Previous Study

The genes studied here were identified from a previous microarray study in males where their transcription was shown to slightly increase (~2 fold on average) in response to selection in the presence of infection [1]. The expression of several of these genes (PGRP-SD, Sr-CI, TepII, eater, Bc) was also confirmed in males using qRT-PCR. In general selected females demonstrate increases in transcription of the same immunity genes (Figure 1) with the exception of TepII and Bc. The magnitude of the female transcriptional response to selection also appears greater, but direct comparisons of expression values between males and females are likely to be confounded by their variation in size and cell count (in particular with respect to the embryo) and any potential sex bias in the reference gene.

## Discussion

In contrast to expectation, we demonstrated that selection either produced similar investments into constitutive and infection-induced transcription (dros4) or invested preferentially in the inducible response (PGRP-SB1, DiptB, Sr-CI, eater, Bc). For PGRP-SB1, DiptB and dros4 heightened inducible expression should confer improved humoral immunity [3]. A failure to see selection induced increases in constitutive expression of these genes may be explained either by pleiotropic or energetic costs or a decoupling of expression between larval and adult stages. Recent work examining the constitutive expression of the immunity genes, Dipt and dro, has shown that they vary with respect to how they are controlled, demonstrating decoupled and coupled patterns of expression, respectively between larvae and adults [22]. If the genes studied here have decoupled control, any changes in the constitutive expression in larvae, may not be present in the adult where we surveyed expression. In contrast, dros4 may be a candidate for coupled control where constitutive changes in larvae are maintained into adulthood.

For the cellular genes, the benefits of increased transcription upon exposure to infection are not clear. Previous genome wide screens of transcription have not revealed induced expression of Sr-CI or eater upon immune challenge with bacteria [4,5,23,24]. Both genes encode transmembrane proteins in insect plasmatocytes that act as scavenger receptors and are required for recognition of pathogens and effective phagocytosis [25,26]. As they are not secreted, it is difficult to understand how their enhanced transcription upon infection would produce immediate benefits with respect to microbial defense. The increased transcription seen here in response to selection may instead serve to directly trigger transcription of other more effective genes. Although the molecular cross talk between the various immunity pathways is not well characterised [27], it has been demonstrated for example that expression of eater is required for complete activation of Defensin [28]. Alternatively, the heightened expression of these genes may not be adaptive. The selected lines may have arrived at genetic solutions where transcription of immunity genes is heightened across the board in a non-specific manner via transcription factors. As above the failure to see increases in constitutive expression in the cellular genes suggests they may be strong candidates for a decoupled model of larval to adult genetic control. In any case, these findings suggest that there is not a global increase in the constitutive expression of cellular immunity genes into adulthood that may explain the rapid development via pleiotropy or the costly tradeoffs seen in host fitness like longevity and egg viability. These costs may instead result from larval phase transcription investments.

The transcription of only one gene, TepII, clearly did not respond to selection. TepII's capacity to respond to bacterial challenge [4,5,13,24] and in particular infection with a particular *P. aeruginosa *strain [23] has been documented previously. There is also evidence that the transcriptional regulation of TepII is at least in partly under the control of the Toll & Imd pathways [4]. More akin to the humoral genes, the encoded protein, is secreted and acts as an opsonin during the process of targeting foreign bacterial invaders for destruction [29]. Production of TepII is required for phagocytosis of bacteria [30] and is involved with wound healing [31]. A failure to improve transcription levels of TepII upon selection may indicate a lack of genetic diversity in the population, although this is unlikely given the response of all other genes. There may also be negative pleiotropic effects associated with heightened transcription. Evidence of TepII's involvement in cellular functions other than immunity is lacking, although it expressed in oocytes [32]. Interestingly, a number of studies have demonstrated strong signatures of positive selection in the protein coding regions of TepII [33-35] where it is thought that variable sequence diversity and the production of multiple isoforms [36] may underpin the ability to bind diverse pathogens. The evolution of such diversity within the gene rather may provide a more effective avenue to improved defense for the insect rather than increased transcription. This is in contrast with a recent study on eater, suggesting that selection for intronic variation rather than protein-coding diversity may have formed the basis of a selective sweep in Drosophila [37].

The fold increases in transcription brought about by selection ranged only from 2 to 3 for all of the various genes. Given that the genes vary widely in their total level of transcription, it is unlikely that the limiters to the degree of transcriptional improvement can be ascribed to cost. Instead it suggests that a shared evolutionary mechanism for discrete increases in transcriptional output may be operating for multiple genes or at least independently at least once for each immune pathway represented here. One possible model includes an evolved increase in the multiplicity of *cis*-regulatory regions [38,39] as that has been documented previously for yeast [40], *Drosophila *[41] and *C. elegans *[42].

## Competing interests

The authors declare that they have no competing interests.

## Authors' contributions

YHY and EAM designed the study. YHY carried out the experimental work. YHY and EAM analyzed the data and drafted the manuscript. Both YHY and EAM read and approved the final manuscript.
